# The effect of heteroscedasticity on the prediction efficiency of genome-wide polygenic score for body mass index

**DOI:** 10.3389/fgene.2022.1025568

**Published:** 2022-11-07

**Authors:** Eun Ju Baek, Hae-Un Jung, Ju Yeon Chung, Hye In Jung, Shin Young Kwon, Ji Eun Lim, Han Kyul Kim, Ji-One Kang, Bermseok Oh

**Affiliations:** ^1^ Department of Biomedical Science, Graduate School, Kyung Hee University, Seoul, South Korea; ^2^ Department of Biochemistry and Molecular Biology, School of Medicine, Kyung Hee University, Seoul, South Korea

**Keywords:** heteroscedasticity, body mass index, prediction efficiency, gene-environment interaction, genome-wide polygenic risk score

## Abstract

Globally, more than 1.9 billion adults are overweight. Thus, obesity is a serious public health issue. Moreover, obesity is a major risk factor for diabetes mellitus, coronary heart disease, and cardiovascular disease. Recently, GWAS examining obesity and body mass index (BMI) have increasingly unveiled many aspects of the genetic architecture of obesity and BMI. Information on genome-wide genetic variants has been used to estimate the genome-wide polygenic score (GPS) for a personalized prediction of obesity. However, the prediction power of GPS is affected by various factors, including the unequal variance in the distribution of a phenotype, known as heteroscedasticity. Here, we calculated a GPS for BMI using LDpred2, which was based on the BMI GWAS summary statistics from a European meta-analysis. Then, we tested the GPS in 354,761 European samples from the UK Biobank and found an effective prediction power of the GPS on BMI. To study a change in the variance of BMI, we investigated the heteroscedasticity of BMI across the GPS *via* graphical and statistical methods. We also studied the homoscedastic samples for BMI compared to the heteroscedastic sample, randomly selecting samples with various standard deviations of BMI residuals. Further, we examined the effect of the genetic interaction of GPS with environment (GPS×E) on the heteroscedasticity of BMI. We observed the changing variance (i.e., heteroscedasticity) of BMI along the GPS. The heteroscedasticity of BMI was confirmed by both the Breusch-Pagan test and the Score test. Compared to the heteroscedastic sample, the homoscedastic samples from small standard deviation of BMI residuals showed a decreased heteroscedasticity and an improved prediction accuracy, suggesting a quantitatively negative correlation between the phenotypic heteroscedasticity and the prediction accuracy of GPS. To further test the effects of the GPS×E on heteroscedasticity, first we tested the genetic interactions of the GPS with 21 environments and found 8 significant GPS×E interactions on BMI. However, the heteroscedasticity of BMI was not ameliorated after adjusting for the GPS×E interactions. Taken together, our findings suggest that the heteroscedasticity of BMI exists along the GPS and is not affected by the GPS×E interaction.

## Introduction

Obesity is a serious public health issue, as it is a major risk factor for diabetes mellitus, coronary heart disease, and cardiovascular disease (Visscher and Seidell, 2001). In 2016, more than 1.9 billion adults were overweight globally, and among them, over 650 million were obese (Mirzaei et al., 2014; World Health Organization, 2020). Moreover, the estimated health care cost driven by the risk factor of obesity was a staggering 480 billion dollars in the United States in 2018, and it continues to rise (Waters and Graf, 2020).

Obesity is typical of a polygenic phenotype and hence influenced by various genetic variants (Silventoinen et al., 2016; O'Connor, 2021). Recent GWAS on obesity and body mass index (BMI) have increasingly unveiled many aspects of the genetic architecture of obesity and BMI (Speliotes et al., 2010; Visscher et al., 2012; Locke et al., 2015; Yengo et al., 2018). As a result, the genome-wide SNP-heritability of BMI was estimated to be from 29% to 44% in the European samples (Hou et al., 2019).

For many common diseases, polygenic inheritance, consisting of multiple genetic variants, plays a greater role than rare monogenic mutations (Khera et al., 2018). Given that, genome-wide polygenic scores (GPSs) for various diseases have been estimated to identify individuals at a clinically increased risk and are expected to contribute to the personalized prediction and prevention of polygenic diseases (Chatterjee et al., 2016; Khera et al., 2018). LDpred is a popular method for deriving GPS based on GWAS summary statistics and a linkage disequilibrium (LD) matrix (Vilhjalmsson et al., 2015). Recently, LDpred2, a newer version of LDpred, was developed, and its computational efficiency is markedly improved and its predictive power is higher than LDpred (Prive et al., 2020). Using LDpred, Khera et al. (2019) created a GPS for BMI and observed a great gradient in weight and the risk of severe obesity across GPS deciles among middle-aged adults (Locke et al., 2015; Khera et al., 2019; Prive et al., 2020).

In general, GPS has been tested using linear regression models between GPS and a phenotype (Khera et al., 2019; Sulc et al., 2020). For linear regression, the two critical assumptions for the data distribution are normality and homoscedasticity (i.e., the equal variance of a phenotype) (Yang et al., 2019). The importance of normality has been enough emphasized when fitting linear regression models, but the importance of homoscedasticity has been often overlooked (Yang et al., 2019). When homoscedasticity is violated, heteroscedasticity occurs in the distribution of a phenotype when the phenotype is fitted in the linear regression model, such that the variance of an individual’s phenotype changes depending on the genotype. The increased degree of heteroscedasticity causes type I errors, yielding a higher rate of false positives (Yang et al., 2019). In particular, when GPS is fitted in a linear regression model, the effect of heteroscedasticity on the prediction efficiency of GPS is not clearly understood yet.

For the unequal variance of a phenotype according to genotypes, there is evidence across different species, such that the variance of phenotypes is genotype dependent (Wolc et al., 2009; Jimenez-Gomez et al., 2011). For example, the *FTO* variant rs7202116 is associated with a 7% difference in variance in BMI between individuals that are homozygous for different alleles (Yang et al., 2012). This may reflect that individuals carrying only one of the alleles may more easily lose or gain weight, whereas individuals with the other allele may have a more stable BMI. The difference in the variance observed for the *FTO* variant has been suggested to be due to different responses to activity levels and other lifestyle factors (Yang et al., 2012; Rask-Andersen et al., 2017). The variance quantitative trait locus (vQTL) analysis has provided empirical evidence for the genetic control of phenotypic variance (Tyrrell et al., 2017). Using the vQTL, Wang et al. (2019) suggested that the gene-environment interaction (G×E) effect of a genetic variant on a quantitative trait could lead to the phenotypic heteroscedasticity of a trait among individuals carrying different genetic alleles (Wang et al., 2019). For BMI, a genetic risk score (GRS) of 376 variants explains 5.2% of the trait variance, and the interaction between GRS and environment (GRS×E) contributes an additional 1.9% (Rask-Andersen et al., 2017; Sulc et al., 2020). On the other hand, it is suggested that the heteroscedastic nature of BMI is most likely due to a transformation and not driven by the GRS×E interaction (Sulc et al., 2020).

In this study, we constructed a GPS *via* LDpred2 using BMI GWAS summary statistics (1,342,646 SNPs) from a European meta-analysis (Locke et al., 2015). When the BMI GPS was validated on 275,809 European samples from the UK Biobank (UKB), we demonstrated the existence of heteroscedasticity of BMI across GPS percentiles. Using the Breusch-Pagan (BP) test and the Score test, we confirmed the heteroscedasticity of BMI across GPS. Further, we showed that using a homoscedastic (or less heteroscedastic) sample, the decreased heteroscedasticity led to an improved prediction efficiency of GPS, suggesting a quantitatively negative relationship between the heteroscedasticity and the prediction accuracy. Further, we investigated the effect of the GPS×E interaction on the heteroscedasticity of BMI. Among 21 environmental factors, we found 8 significant GPS×E interactions on BMI using 216,103 European samples from the UKB. Then we studied whether the heteroscedasticity changed after adjustments for each of 8 GPS×E interactions using BP and Score testes.

## Materials and methods

### Study population and design

The UKB is a population-based cohort that recruited over 487,409 individuals aged 40–69 years in the United Kingdom during 2006–2010 (Collins, 2012). For quality control, we used the following filter parameters from the Neale lab (https://github.com/Nealelab/UK_Biobank_GWAS): a principal component analysis (PCA) to filter for the selection of unrelated samples; a sex chromosome filter to remove aneuploidy; a principal components (PCs) filter for European sample selection to determine British ancestry; and filters for the selection of self-reported ‘white British’, ‘Irish’, and ‘White’. We then selected 354,761 unrelated European samples from the UKB participants, of which 10,000 samples were used for a linkage disequilibrium (LD) reference, 68,952 samples were used to calculate the candidate GPSs (test set), and 275,809 samples were used to validate a GPS with selected parameters (validation set) (Table 1). Access to the UKB data was granted under application no. 48422 “Gene-environment interaction study on obesity, body mass index, and waist circumference”.

**TABLE 1:** Characteristics of the UK Biobank samples.

		Total	Test set (20%)	Validation set (80%)
Sample size (N)	—	344,761	68,952	275,809
Age (years)	—	56.75 ± 7.98	56.77 ± 7.97	56.74 ± 7.98
Sex (%)	Female	185,664 (53.85%)	37,023 (53.69%)	148,641 (53.89%)
	Male	159,097 (46.15%)	31,929 (46.31%)	127,168 (46.11%)
BMI (kg/m^2^)	—	27.38 ± 4.76	27.37 ± 4.77	27.29 ± 4.76

Data, mean ± standard deviation (SD) or N (%), unless otherwise stated.

BMI, body mass index.

### Ethics approval and consent to participate

The UKB was granted ethical approval to collect data from participants by the North West Multicentre Research Ethics Committee, the National Information Governance Board for Health & Social Care, and the Community Health Index Advisory Group.

### Genotype data

At baseline, imputation data for 93,095,623 SNPs were available for 487,409 participants using the UKB Axiom Array and the UK BiLEVE Axiom Array from Affymetrix (Sudlow et al., 2015). Genotyping imputation was performed using the UK10K Project and 1,000 Genome Project Phase 3 reference panels (UK10K Consortium et al., 2015). Quality control was performed based on the following exclusion criteria using PLINK v.1.90: SNPs with missing genotype call rates >0.05; minor allele frequency (MAF) < 0.01; and *p*-value for Hardy-Weinberg equilibrium test <1.00 × 10^−6^ (Purcell et al., 2007). A total of 5,664,578 SNPs were retained for further analysis.

### Phenotype data and environmental variables

The BMI value was determined using the height and weight measured during the initial UKB assessment center visit. Individuals with missing BMI data were discarded from the analysis (Table 1).

Based on a previous report (Young et al., 2016), we included 21 environmental variables for genetic interaction studies, discarding individuals with missing environmental variables. “Prefer not to answer” and “I do not know” were set as “missing” in our analyses (Supplementary Table S1). The 21 environmental variables included 12 dietary variables (beef intake, bread intake, cheese intake, cooked vegetable intake, lamb/mutton intake, non-oily fish intake, oily fish intake, port intake, poultry intake, processed meat intake, salt added to food, and tea intake), 6 lifestyle variables (alcohol intake frequency, sleep duration, sleep duration residual squared, smoking status, townsend deprivation index (TDI), and time spent watching television (TV)), and 3 physical activity variables (number of days/week walked 10+ minutes, number of days/week moderate physical activity 10+ minutes, and number of days/week vigorous physical activity 10+ minutes). For ‘sleep duration residual squared’, for each individual, we calculated, the squared deviations from the mean sleep duration.

### Creation of genome-wide polygenic score

Candidate GPSs were derived using the LDPred2 computational algorithm that is based on a Bayesian approach using an LD matrix and GWAS summary statistics, implemented in the R package bigsnpr (Prive et al., 2020). LDpred2 provides more hyper-parameters (102 grids) than LDpred1 (7 grids). Among the models, the infinitesimal model assumes that all the genetic variants are causal. The grid model tunes the hyper-parameters of SNP heritability (*h* ^*2*^), the proportion of causal variants (*p*), and the optional sparsity to reweight the variant effect to the phenotype. The SNP heritability (*h* ^*2*^) was estimated using LD Score regression between summary statistics and LD score from the European-ancestry samples in the 1000 Genomes Project (Bulik-Sullivan et al., 2015). The grid sparsity is an option to reduce SNPs by fitting some effects to zero, providing a sparse vector of the effects. Hence, the sparsity may reduce the computing time without losing the predictive accuracy (Prive et al., 2020).

To calculate a GPS, we obtained the GWAS summary statistics on the BMI from the European meta-analysis (Locke et al., 2015). For the LD reference, using 10,000 European samples, we computed the LD correlation matrix among 1,342,646 SNPs consisting of a common set of both HapMap3 variants and the BMI GWAS meta-summary statistics (International HapMap 3 Consortium et al., 2010; Yengo et al., 2018; Prive et al., 2020). Using 344,761 European samples, we estimated 3 models (infinitesimal, grid, and grid sparse model) to construct the GPS. For the infinitesimal model, where all the markers are causal, we estimated the infinitesimal model using a total of 344,761 participants. For the grid models, we tested a total of 102 grid models of hyper-parameters, consisting of *p* (proportion of causal variants, 17 values from 0.0001 to 1), *h* ^2^LDSC (heritability, 3 values at 0.1027, 0.1467, and 0.2054), and the existence of sparsity. Using 68,952 European samples (test set) for the grid models, the best model was determined by the adjusted *R* ^2^ (the explained phenotypic variance) *via* a linear regression model between the GPS and a phenotype, which was adjusted for age, sex, array, and 10 PCs (BMI ∼ GPS + age + sex + array +10 PCs) (Table 1, Supplementary Table S2, and Supplementary Figure S1). Once the optimal hyper-parameters were set, 275,809 European participants were used as a validation set (Table 1, Supplementary Table S2, and Supplementary Table S3).

To estimate the prediction accuracy of the GPSs, we used the adjusted *R* ^2^ and mean squared error (MSE) in a linear regression model between GPS and BMI and adjusted for age, sex, array, and 10 PCs.

### Statistical analysis

We performed an association analysis *via* linear regression, and a variance test, such as the Fligner Killeen test (FK-test), scatter plotting, and density plotting using the R stats package (version 4.0.2; www.r-project.org). To draw the scatter plot and density plot, the “ggplot2” package was used. Linear regression modeling was used to estimate the prediction accuracy of GPS (BMI ∼ GPS + age + sex + array +10 PCs) and the effect of gene-environment interaction (GPS×E) on BMI (BMI ∼ GPS + E + GPS×E + age + sex + array +10 PCs). When the heteroscedasticity existed, as a sensitivity analysis, we performed the robust regression analysis by calculating the robust (or “White”) standard error of the GPS×E interaction effects, using the vce (robust) option in STATA (Cribari-Neto, 2004; Tyrrell et al., 2017).

The Breusch-Pagan (BP) and Score tests were used to assess the heteroscedasticity of BMI across the GPS. The FK-test was used to compare the variances between the GPS quintile (Q) subgroups. To compare the interaction effect size between Q1 and Q5, we computed *p*-values (*P*_difference_) by testing for difference between Q1 and Q5 beta-estimates with their corresponding standard errors SE1 and SE5 using the t statistic (Randall et al., 2013).

For quantifying the relationship between the heteroscedasticity of BMI and the prediction accuracy of GPS, linear regression analyses were performed using randomly selected samples with various SD cutoffs from our UKB validation set (275,809 participants). First, 10,000 participants were randomly selected from each group with a SD cutoff (from 0.25 to 2 by 0.25 increment) of the BMI residual mean and this random selection was repeated in each group until 100 times. Then, the linear regression model was adjusted for age, sex, array, and 10 PCs. In each model, *R* ^2^ and MSE were estimated, and BP and Score tests were performed. Then, the correlation analysis between the prediction accuracy (*R* ^2^ or MSE) and heteroscedasticity statistics (χ^2^ of BP or Score test) was performed using the statistics from all 800 models.

## Results

### Construction of GPS for BMI

To estimate the GPS, we used the GWAS summary statistics on BMI from the European meta-analysis (Locke et al., 2015) and the LD correlation matrix. We then tested 3 models (infinitesimal, grid, and grid sparse model) to construct the GPS using 68,952 unrelated Europeans from the UKB participants (the infinitesimal model used both the test and validation sets). The best model for GPS was the grid model (for grid: *p* = 0.18, *h* ^*2*^ = 0.2054, and *R* ^2^ = 0.3192), which was based on the *R* ^2^ when fitting to a linear regression model (Supplementary Table S2).

**FIGURE 1:**
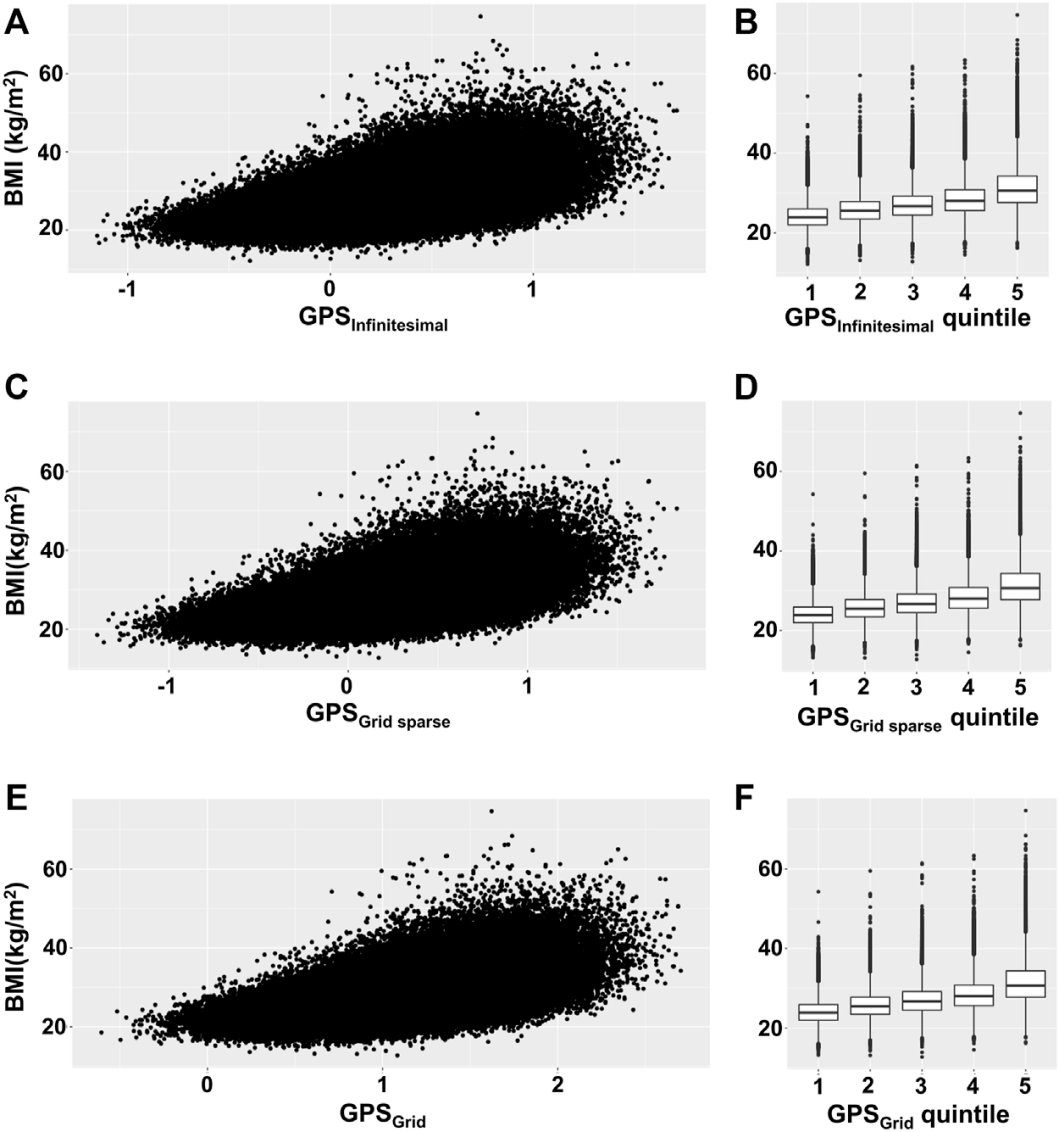
Scatterplot and box plot of BMI across GPS. **(A,C,E)** BMI was plotted against GPS for each GPS model, using the validation set. **(B,D,F)** The mean and variance of BMI were depicted by box plot per GPS quintile subgroup. **(A,B)** Infinitesimal model using both test and validation sets, **(C,D)** grid sparse model using only the validation set, and **(E,F)** grid model using only the validation set.

To visualize the relationship between BMI and GPS, we depicted the BMI scatter plots for the 3 GPS models (infinitesimal, grid, and grid sparse) using the validation set (the infinitesimal model used both the test and validation sets). We observed that the BMI variance tended to increase across the GPS in all 3 models (Figures 1A,C,E). To estimate how much the variance changed, we binned GPS into 5 quintiles (Q) subgroups and plotted the mean value and the BMI variance per quintile group (Figures 1B,D,F; Table 2). These plots showed that the BMI variance increased according to the GPS quintiles.

**TABLE 2:** The mean, SD, and variance of BMI for each GPS quintile group.

	GPS quintile	Mean (kg/m^2^)	SD	Variance
GPS_Infinitesimal_	Q1	24.16	3.11	9.64
	Q2	25.84	3.46	11.97
	Q3	27.08	3.84	14.74
	Q4	28.49	4.27	18.24
	Q5	31.35	5.37	28.88
GPS_Grid sparse_	Q1	24.07	3.04	9.27
	Q2	25.81	3.41	11.64
	Q3	27.07	3.79	14.33
	Q4	28.51	4.21	17.72
	Q5	31.46	5.36	28.74
GPS_Grid_	Q1	24.07	3.04	9.21
	Q2	25.80	3.41	11.63
	Q3	27.07	3.78	14.27
	Q4	28.51	4.19	17.58
	Q5	31.49	5.36	28.75

SD, standard deviation.

GPS_Infinitesimal_ consists of total samples, GPS_Grid, sparse_ and GPS_Grid_, consists of validation set samples.

To see the distribution of the obese *versus* normal subjects, we depicted the population density plot for each GPS model. The mean GPS shifted to the right for the obese subjects (BMI ≥30 kg/m^2^) *versus* the normal subjects (BMI <30 kg/m^2^), suggesting that all 3 GPS models distinguished between the obese and normal groups (Supplementary Figure S2). All 3 GPS models showed significant associations with BMI based on the linear regression model that was adjusted for age, sex, genotyping array, and 10 PCs (*p*-value < 2.00 × 10^−16^) (Supplementary Table S3). Consistently, when using the test and validation sets, the grid model for GPS showed the best *R* ^2^ and the explained phenotypic variance compared to the null model (R^2^ Grid = 0.3216, R^2^Grid sparse = 0.3185, and R^2^Infinitesimal = 0.3022). Hence, this Grid model was used in the following studies (Supplementary Tables S2, S3).

### Heteroscedasticity of BMI across GPS

**FIGURE 2:**
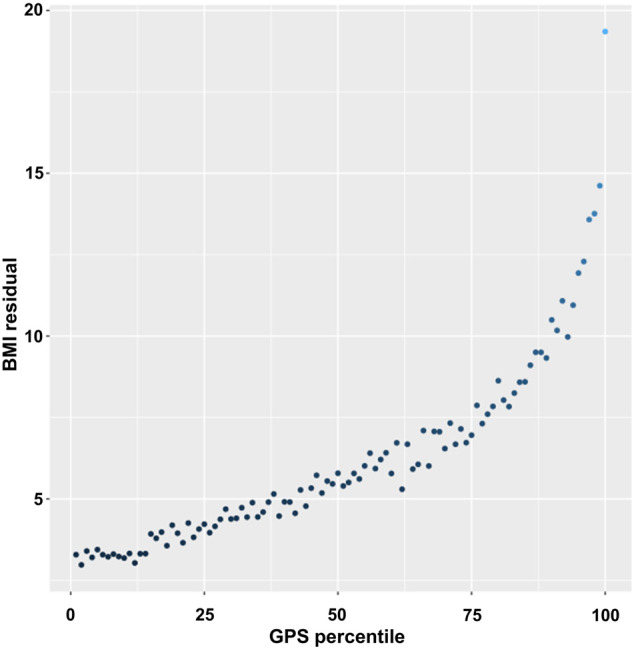
Plot of BMI residuals by GPS percentile. Samples were binned into 100 equally sized groups by GPS. In each group, the mean of residuals of BMI was calculated and plotted against the mean value of GPS in the GPS percentile group.

To better assess the changes in the BMI variance, we estimated the residuals of the individuals (the difference between a set of observed and predicted values) using linear regression for BMI and adjusted for age, sex, genotyping array, and 10 PCs and then plotted the averaged BMI residuals according to the GPS percentiles (Figure 2). The averaged residuals plot showed a great increase in BMI residuals as the GPS increased, suggesting a violation of homoscedasticity, i.e., heteroscedasticity (Figure 2).

For a statistical assessment of heteroscedasticity, we performed the BP and Score tests on the GPS total and quintile subgroups. Both tests statistically confirmed a heteroscedastic inequality in the residuals according to the increasing GPS, with the highest heteroscedasticity in Q5 and the smallest in Q1, among the 5 quintiles (Table 3) (Rosopa et al., 2013). We then also assessed whether there is variation in the residuals between GPS quintile groups *via* the FK test. The results showed statistically significant discrepancies in the residuals between the GPS Q1 and the other 4 quintile groups (Q2-5) (Supplementary Table S4). We then assessed the prediction accuracy of GPS *via* the MSE of the linear regression model. The results showed an increasing MSE (i.e., a decreasing accuracy) as the GPS quintiles increased (Table 3). The residuals plot and heteroscedasticity analyses suggested that BMI followed a heteroscedastic distribution across the GPS subgroups and the increased heteroscedasticity might lead to GPS imprecision.

**TABLE 3:** The heteroscedasticity of BMI according to GPS quintile.

Group^a^	Number	Variance^b^	MSE	Breusch-Pagan test		Score test	
				χ^2^	*p*-value	χ^2^	*p*-value
Total	275,809	22.63	2.96	19288.15	<1.00E-300	8104.81	<1.00E-300
Q1	55,162	9.21	2.18	48.66	3.05E-12	26.25	3.00E-07
Q2	55,162	11.63	2.55	147.63	5.72E-34	73.61	9.50E-18
Q3	55,162	14.27	2.84	311.19	1.20E-69	140.36	2.22E-32
Q4	55,162	17.58	3.17	100.63	1.11E-23	49.59	1.90E-12
Q5	55,161	28.75	3.88	1,198.58	1.24E-262	654.88	1.94E-144

MSE, mean squared error.

^a^ BMI ∼ GPS + age + sex + array +10 PCs.

^b^ Indicates the variance of BMI.

The analysis was performed using samples from validation set.

In general, when fitting linear regression models, the normality of a phenotype is a critical factor for achieving better power performance and less inflated type I errors (Chien, 2020). In addition, the normality of a phenotype seems to affect the heteroscedasticity (Sulc et al., 2020). To further test whether the skewed (or non-normal) distribution of the phenotype drives the heteroscedasticity, we performed heteroscedasticity analyses (i.e., BP and Score tests) following normal transformation of BMI using 3 different methods [log-, Box-Cox, and rank-based inverse normal transformation (INT)] (Supplementary Figures S3, S4) (Buzkova, 2013; Koenker, 2022; Chien, 2020). The association analyses showed that all 3 normalizations of BMI improved the prediction accuracy of GPS based on *R* ^2^ and MSE, compared to non-normalized BMI, except that the MSE for logBMI worsened (Supplementary Table S5). The heteroscedasticity greatly decreased when using normalized BMIs compared to non-normalized BMI, of which the INT transformation almost removed the heteroscedasticity, the Box-Cox method greatly decreased the heteroscedasticity, and the log-transformation weakly decreased it (Supplementary Table S5). Our findings suggest that for BMI, all 3 normalizations are effective to correct the heteroscedasticity and accordingly to improve the prediction accuracy of BMI GPS (Nwakuya and Nwabueze, 2018).

### Analysis of homoscedastic samples for BMI across GPS

To better understand the heteroscedasticity, we examined the homoscedastic (or less heteroscedastic) samples in relation to the prediction efficiency. Two different homoscedastic (or less heteroscedastic) samples were extracted from the samples with 1 standard deviation (SD) and 2SD of the residual mean of the linear regression for BMI (Supplementary Figurers S5A,D). The selected 1SD and 2SD samples were binned into 5 quintile subgroups. The plots of the mean and variance for BMI showed a smaller variation for the 1SD and 2SD samples for BMI according to the GPS quintiles (Supplementary Figurers S5B,E). Consistently, the plots of the BMI residuals showed a reduced increase for the 2SD sample and were almost unchanged for the 1SD across the GPS percentiles (Supplementary Figurers S5C,F). Both the BP and Score tests showed consistent results, such that the degree of heteroscedasticity decreased in the 2SD sample and dramatically dropped in the 1SD sample, suggesting the 2SD sample was a less heteroscedastic sample and 1SD was a homoscedastic sample (Supplementary Table S5).

We then assessed the prediction accuracy of GPS *via* the adjusted *R* ^2^ or MSE of the linear regression model. The *R* ^2^ was 0.6095 for the 1SD samples (N = 201,568) and 0.3746 for the 2SD samples (N = 263,310), showing an increase greater than 0.3216 for the total samples (N = 275,809) (Supplementary Table S5). Consistent with the *R* ^2^, the MSE decreased in the 2SD sample compared to the total samples and more greatly decreased for the 1SD (Supplementary Table S5). These results implied that the increased degree of heteroscedasticity might adversely affect the prediction efficiency of GPS.

**FIGURE 3:**
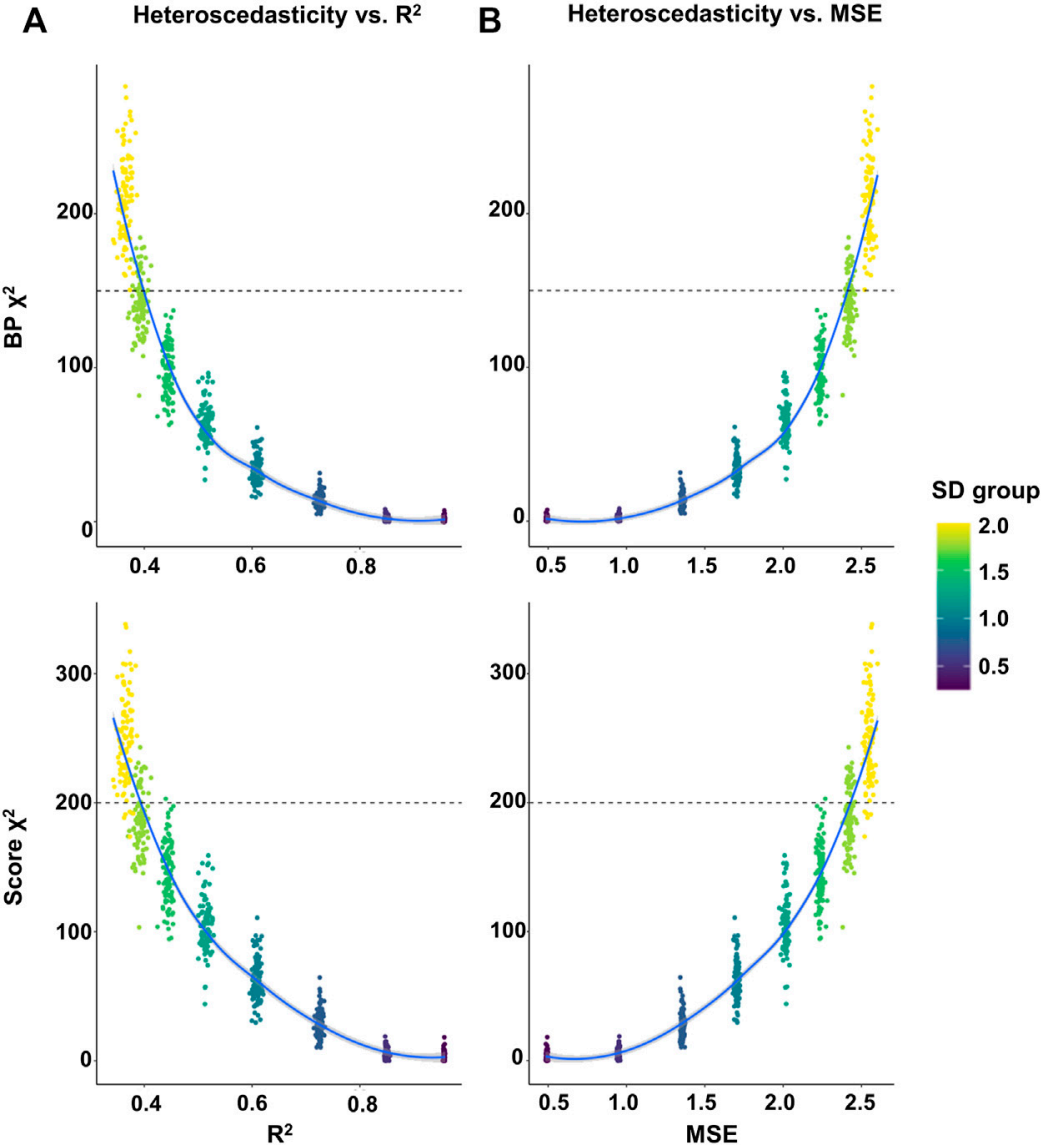
Plot of the heteroscedasticity *versus* the prediction accuracy of GPS in randomly selected samples. Linear regression analyses on GPS were performed using randomly selected samples with various SD cutoffs (from 0.25 to 2 by 0.25 increment) in the UKB validation set. The random selection of 10,000 samples were repeated 100 times and each sample was used for a linear regression analysis. **(A,B)** The heteroscedasticity (χ^2^ of BP in the upper panel and Score test in the lower panel) in each sample was plotted against the prediction accuracy (*R* ^2^ in A and MSE in B) of GPS.

To further quantify the relationship between the phenotypic heteroscedasticity and the prediction accuracy of GPS, we performed linear regression analyses on GPS using randomly selected samples with various SD cutoffs (from 0.25 to 2 by 0.25 increment) from our UKB validation set (275,809 participants). The results showed that as the SD cutoffs increased, the averaged prediction accuracy (*R* ^2^ and MSE) of GPS decreased and the averaged heteroscedasticity (χ^2^ of BP and Score test) increased (Supplementary Table S6). Next, the correlation analysis between the prediction accuracy (*R* ^2^ or MSE) and heteroscedasticity statistics (χ^2^ of BP and Score test) suggested a strong negative correlation between them (*R* ^2^ vs χ^2^BP = -0.86, *p* = 4.05E-232; *R* ^2^ vs χ^2^score = -0.90, *p* = 8.99E-296; MSE vs χ^2^BP = 0.86, *p* = 3.49E-232; MSE vs χ^2^score = 0.90, *p* = 4.07E-293) (Supplementary Table S6). Moreover, the χ^2^ values of heteroscedasticity were plotted against the GPS accuracy. The plots showed an exponential distribution of heteroscedasticity against the accuracy of GPS, suggesting that the prediction accuracy may greatly increase when the degree of heteroscedasticity is low (χ^2^ < 150 for BP; < 200 for Score test) while the accuracy may slightly vary or be unchanged when the heteroscedasticity is too high (χ^2^ > 150 for BP; > 200 for Score test) (Figure 3).

Among the GPS quantile groups from the total heteroscedastic samples, 64.92% of Q1 were normal weight, 31.6% were overweight, and 3.48% were obese, while 7.95% of Q5 were normal, 36.09% were overweight and 55.96% were obese (Supplementary Figure S6). In the Q1 of the 2SD samples, 65.77% were normal weight, 31.99% were overweight, and 2.24% were obese, while in Q5, 6.83% were normal weight, 40.12% were overweight, and 53.06% were obese. In the Q1 of the 1SD samples, 70.61% were normal weight, 29.39% were overweight, and nobody was obese, while in the Q5, 0.02% were normal weight, 41.95% were overweight, and 58.04% were obese (Supplementary Figure S6). Consistent with the high *R* ^2^, the GPS in the 1SD samples targeted a narrower range of obesity than in the total samples, indicating a greater prediction power in the homoscedastic (or less heteroscedastic) sample than in the heteroscedastic sample.

### Heteroscedasticity of BMI attributed to the GPS×E interaction

Since there have been controversies surrounding the role of the G×E interaction in phenotypic heteroscedasticity (Wang et al., 2019; Sulc et al., 2020), we aimed to determine the effect of the GPS×E interaction on the heteroscedasticity of BMI. To select the environmental variables for the GPS×E, we tested associations between BMI and 21 environmental variables that had been previously reported to be associated with BMI (Supplementary Table S7) (Young et al., 2016). Among the 21 variables, 20 showed significant associations between BMI and environmental variables after Bonferroni correction (*p*-value < 0.05/21 = 0.002) (Supplementary Table S7). We then performed 20 GPS×E interaction analyses using a linear regression model that was adjusted for age, sex, genotyping array, and 10 PCs. Of the 20 environmental variables, 15 GPS×E interactions satisfied the statistical significance threshold (*p*-value < 0.05/20 = 0.0025) (Supplementary Table S8). Because heteroscedasticity was present, as a sensitivity analysis, we performed the robust regression analysis, the “White test,” for the interaction studies (Tyrrell et al., 2017). The results showed that 14 GPS×E interactions were still significant *via* the White test, excluding the overriding of the false positives (Supplementary Table S8). As another sensitivity assay, we tested whether the GPS×E interaction effects differed between the GPS Q1 and Q5 groups. The linear regression analyses for the GPS×E interaction were performed for the GPS Q1 and Q5 groups, followed by the stratification analysis between the Q1 and Q5 groups using the GPS×E interaction results for each group. Among the 14 environmental variables, 8 GPS×E interactions met the significance threshold after Bonferroni correction (*p*-value < 0.05/20 = 0.0025; pork intake, processed meat intake, tea intake, alcohol intake frequency, TDI, number of days/week walked 10+ minutes, number of days/week of moderate physical activity 10+ minutes, and number of days/week of vigorous physical activity 10+ minutes) (Supplementary Table S8). Taken together, our results implicated at least 8 significant GPS×E interactions on BMI.

We further studied whether GPS×E interactions are specific to the measured environmental factor or represent a more general pattern of moderation of the total variance in BMI by the environmental factor. We applied the heteroscedastic GPS×E regression model for our BMI GPS on BMI including the GPS×E term. The results showed that 6 GPS×E interactions (processed meat intake, alcohol intake, TDI, number of days/week walked 10+ minutes, and number of days/week of moderate/vigorous physical activity 10+ minutes) were significantly specific to the environmental factors, while the other 2 interactions (pork and tea intake) might present an environmental heteroscedasticity (Supplementary Table S9). The results of the heteroscedastic GPS×E regression modelling suggest that 2 of 8 GPS×E interactions may not be true positive interactions.

To study a change in the heteroscedastic variance of BMI attributable to the GPS×E interaction, we performed the BP and Score tests *via* linear regression of BMI and GPS after adjusting for each of the 8 GPS×E interactions. The BP and Score tests showed that the heteroscedasticity increased after the adjustment for each of the 8 GPS×E interactions compared to the sample without the adjustment (Supplementary Table S10). These results suggested that the GPS×E interactions might not always contribute to the heteroscedasticity of BMI. Further, we studied whether the adjustment for GPS×E interaction could alter the prediction accuracy of GPS. We found that based on *R* ^2^ and MSE, the prediction accuracy of GPS slightly increased after adjusting for GPS×E interactions (Supplementary Table S10).

## Discussion

We estimated a GPS composed of 1.3 million SNPs from the BMI GWAS meta-summary statistics using LDpred2 (Locke et al., 2015; Prive et al., 2020). We observed that the standard deviations of BMI varied across the GPS percentiles and statistically confirmed the heteroscedasticity. Furthermore, our findings suggested an improved prediction efficiency of GPS in the homoscedastic sample compared to the heteroscedastic sample.

Previous studies have acknowledged the presence of heteroscedasticity in studies using large sample sizes for GWAS (Tyrrell et al., 2017; Sulc et al., 2020). However, those studies mainly focused on the role of heteroscedasticity resulting in false positives for the GRS×E interactions, not on its role in the efficient prediction of the GPS. Therefore, our study is the first to manifest the heteroscedasticity of BMI across GPS and further show the possible modulation of heteroscedasticity on the prediction power of the GPS. We then tested the effect of the GPS×E interaction on heteroscedasticity, because previous studies suggest that G×E interactions may contribute to the heteroscedasticity of phenotypes across different genotypes (Yang et al., 2012; Rask-Andersen et al., 2017; Tyrrell et al., 2017; Wang et al., 2019; Marderstein et al., 2021). However, we found that the heteroscedasticity remained significant after adjusting for the GPS×E interaction. Regarding the controversies over the origin of the phenotypic variation by genotypes, our findings support a previous report that the heteroscedasticity of BMI is not driven by the G×E interaction (Sulc et al., 2020). Despite the increased heteroscedasticity, the prediction accuracy of GPS slightly increased after adjustment for GPS×E interactions. Our previous results using randomly selected samples with various SD cutoffs suggested a negative relationship between the heteroscedasticity and the prediction accuracy of GPS. However, the results after adjusting for GPS×E interactions are inconsistent with the negative correlation between heteroscedasticity and accuracy. We assume that the prediction accuracy of GPS may slightly vary or be unchanged when the heteroscedasticity is too high since the accuracy exponentially decreases as the heteroscedasticity increases. Our sample for studying GPS×E interactions presents extremely high heteroscedasticity. Hence, we presume that our sample may not be an appropriate sample that can differentiate the effect of GPS×E interaction on the prediction accuracy of GPS.

We found at least 8 significant GPS×E interactions for BMI. Previous studies using GRSs based on genome-widely significant BMI-associated variants suggest that the genetic effects on BMI are modulated by various lifestyle factors, such as diet (red/processed meat intake), alcohol intake frequency, usual walking pace, TDI, and moderate/vigorous physical activity (Rask-Andersen et al., 2017; Tyrrell et al., 2017; Ding et al., 2018). Our findings using 8 GPS×E interactions were consistent with previous reports, supporting the modulation of genetic factors on BMI by 4 environmental factors (i.e., diet, alcohol, TDI, and physical activity).

Although we constructed a GPS for BMI with high prediction power and demonstrated the presence of heteroscedasticity, we acknowledge several limitations in our study. First, we studied the heteroscedasticity of BMI across GPS but were not able to identify the possible causes for heteroscedasticity. However, we most likely ruled out the role of the G×E interaction in causing heteroscedasticity. For BMI, rare genetic mutations, such as the loss of functions or rare variants (MAF <0.01), have been reported to contribute considerably to its heritability (Akbari et al., 2021; Wainschtein et al., 2022). The contribution of rare genetic variants to heteroscedasticity and the prediction accuracy is not clearly understood yet. Hence, our hope for identifying the original cause for heteroscedasticity and hence, improving the prediction accuracy of GPS is largely unexplored yet. Second, our study focused on BMI, and thus, caution is noted in applying our results to other traits. Despite an important assumption, heteroscedasticity is often overlooked in linear regression models (Yang et al., 2019). Thus far, the association of phenotypic heteroscedasticity and GPS has not been studied in GPS models for other traits, regarding the prediction power of GPS. We believe that our study provides a good basis for the effect of phenotypic heteroscedasticity on GPS.

GPS is a forefront study that warrants more careful characterization for its prediction power in different backgrounds. We believe that our findings brush away a flow in the crystal and hence, provide a good basis for using GPS for predicting individuals at genetically increased risk.

## Data Availability

The datasets presented in this study can be found in online repositories. The names of the repository/repositories and accession number(s) can be found in the article/Supplementary Material.
