# Supplementary figures and images for: The effect of heteroscedasticity on the prediction efficiency of genome-wide polygenic score for body mass index
Source: Front Genet. 2022 Nov 7;13:1025568. doi: 10.3389/fgene.2022.1025568 (PMC9676478; doi:10.3389/fgene.2022.1025568)

Figure S1.

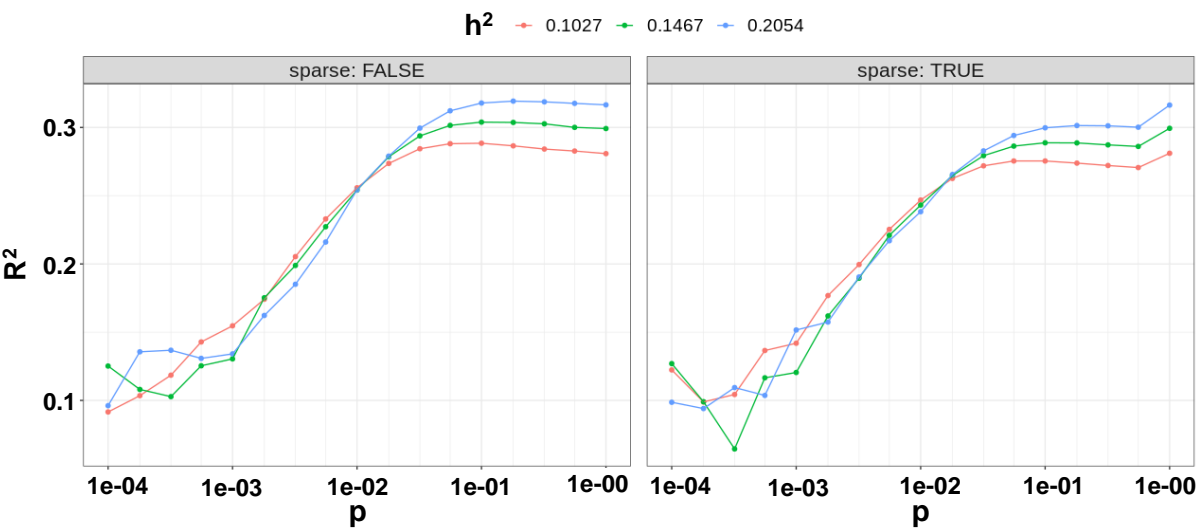

Figure S2.

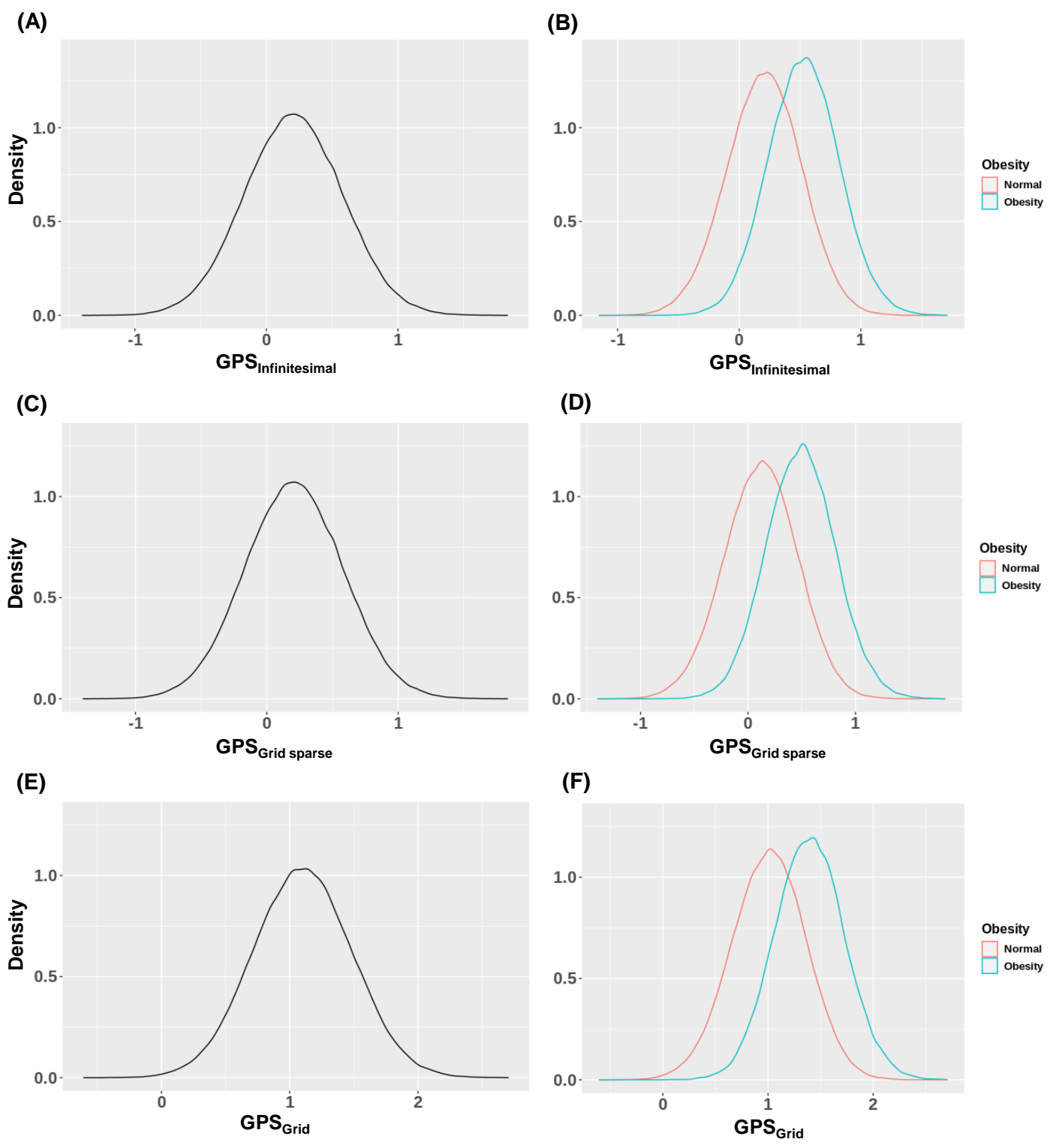

Figure S3.

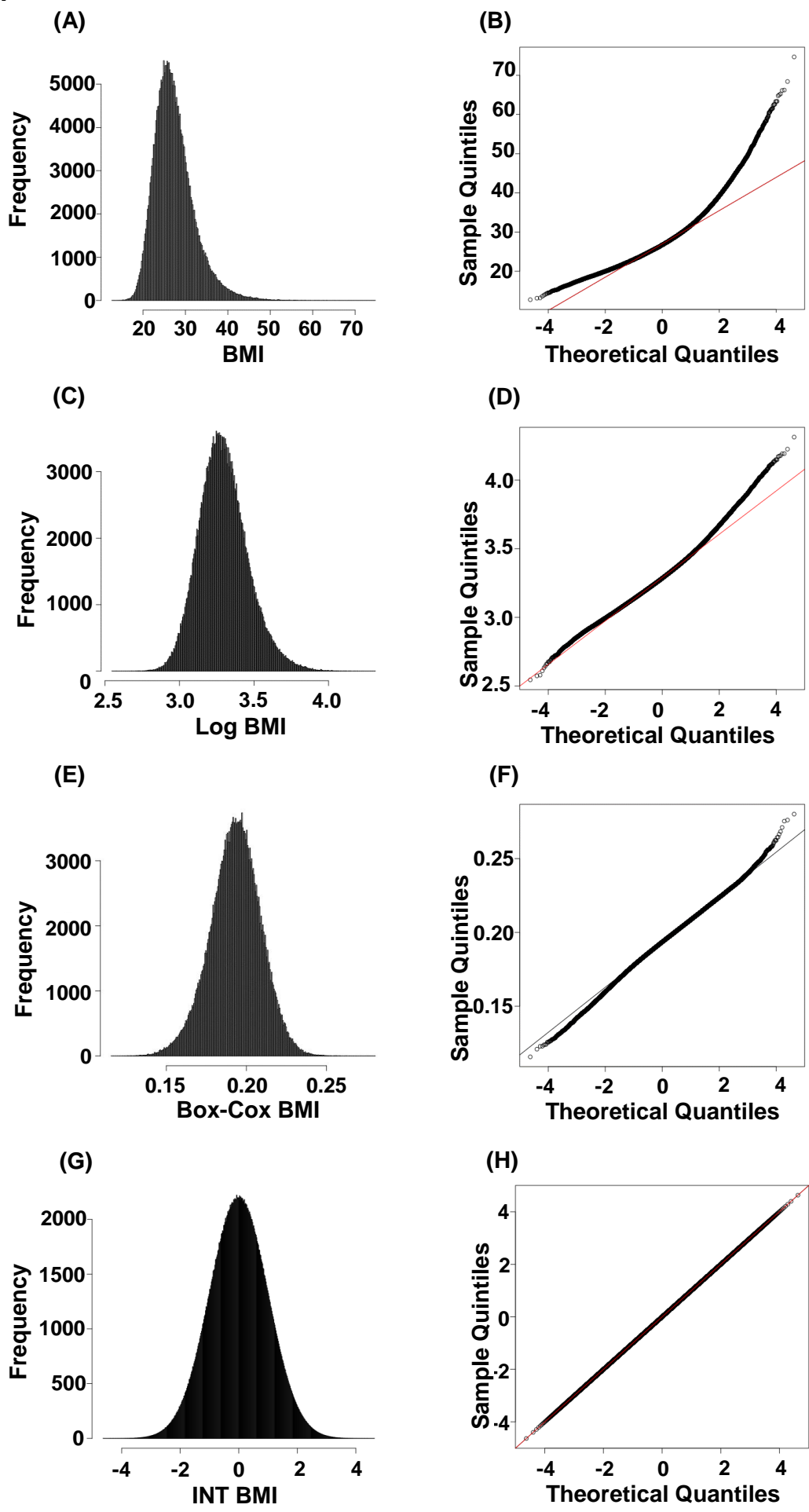

Figure S4.

(A)

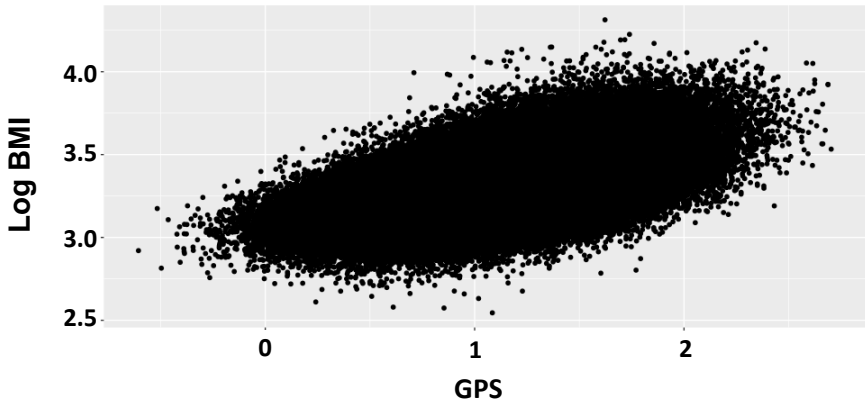

(B)

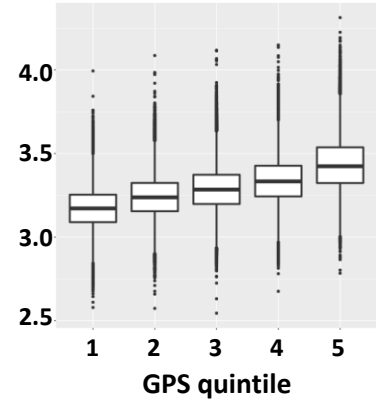

(C)

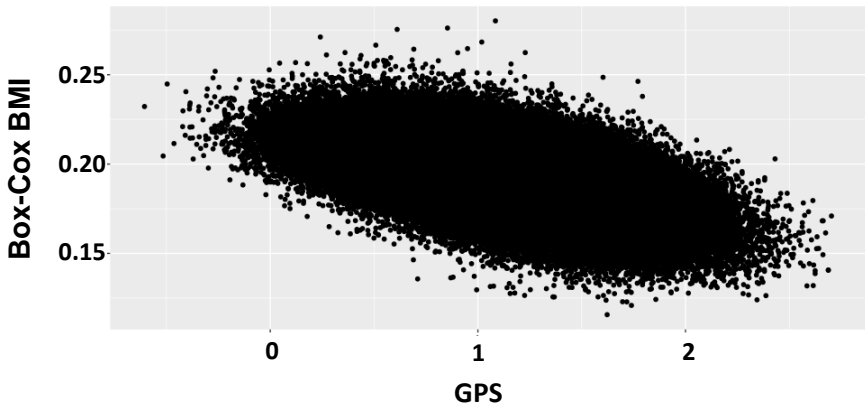

(D)

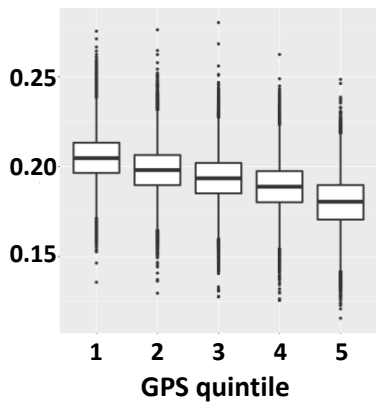

(E)

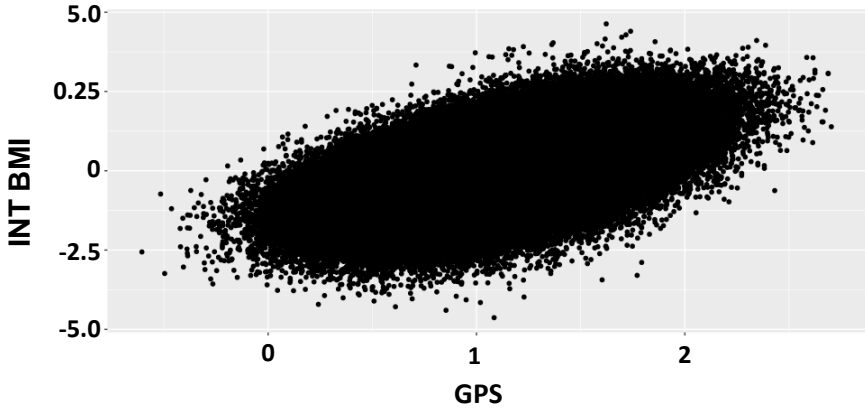

(F)

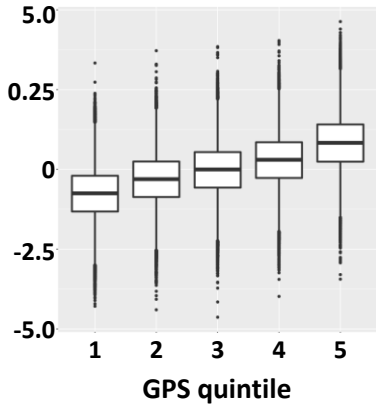

Figure S5.

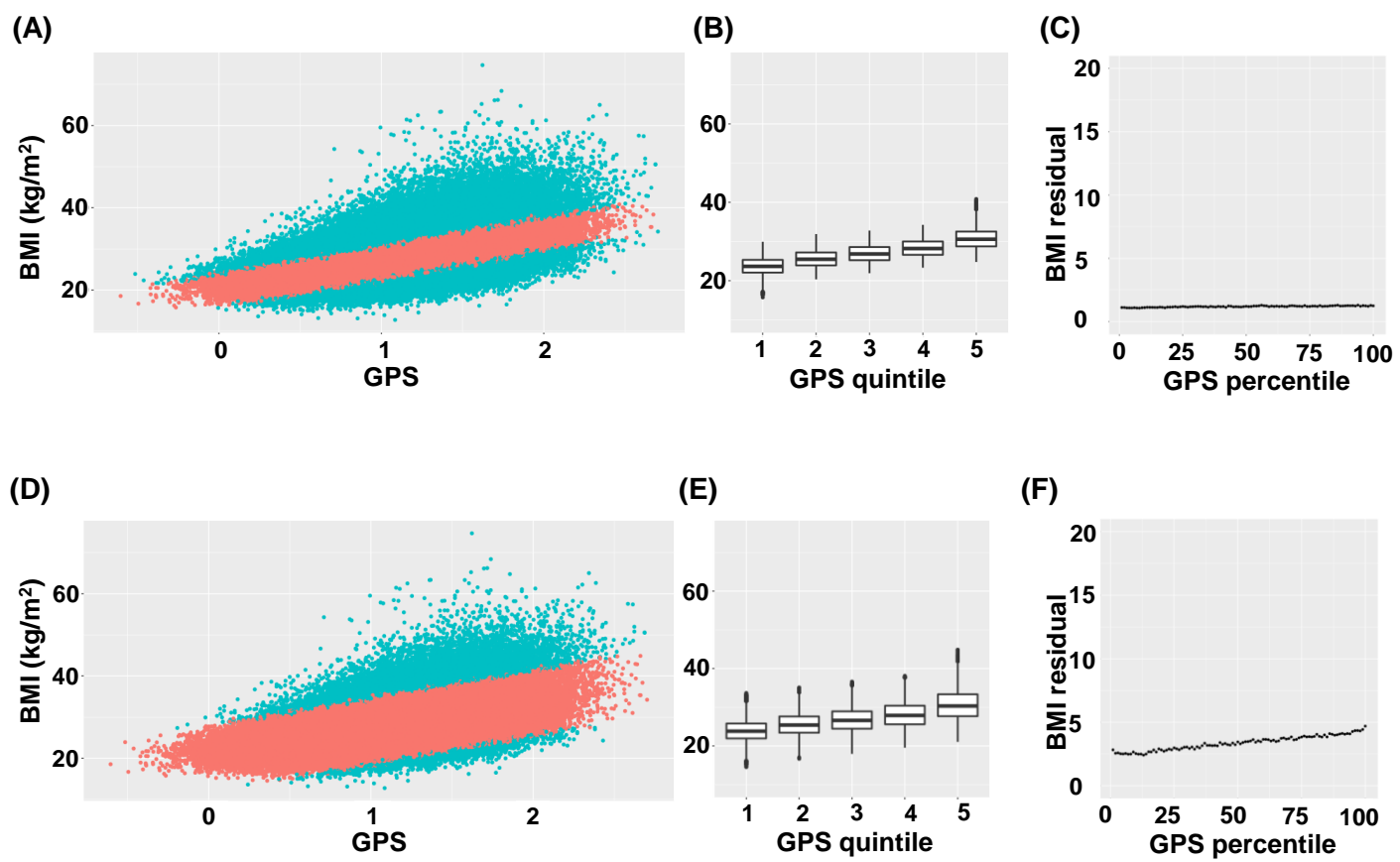

Figure S6.

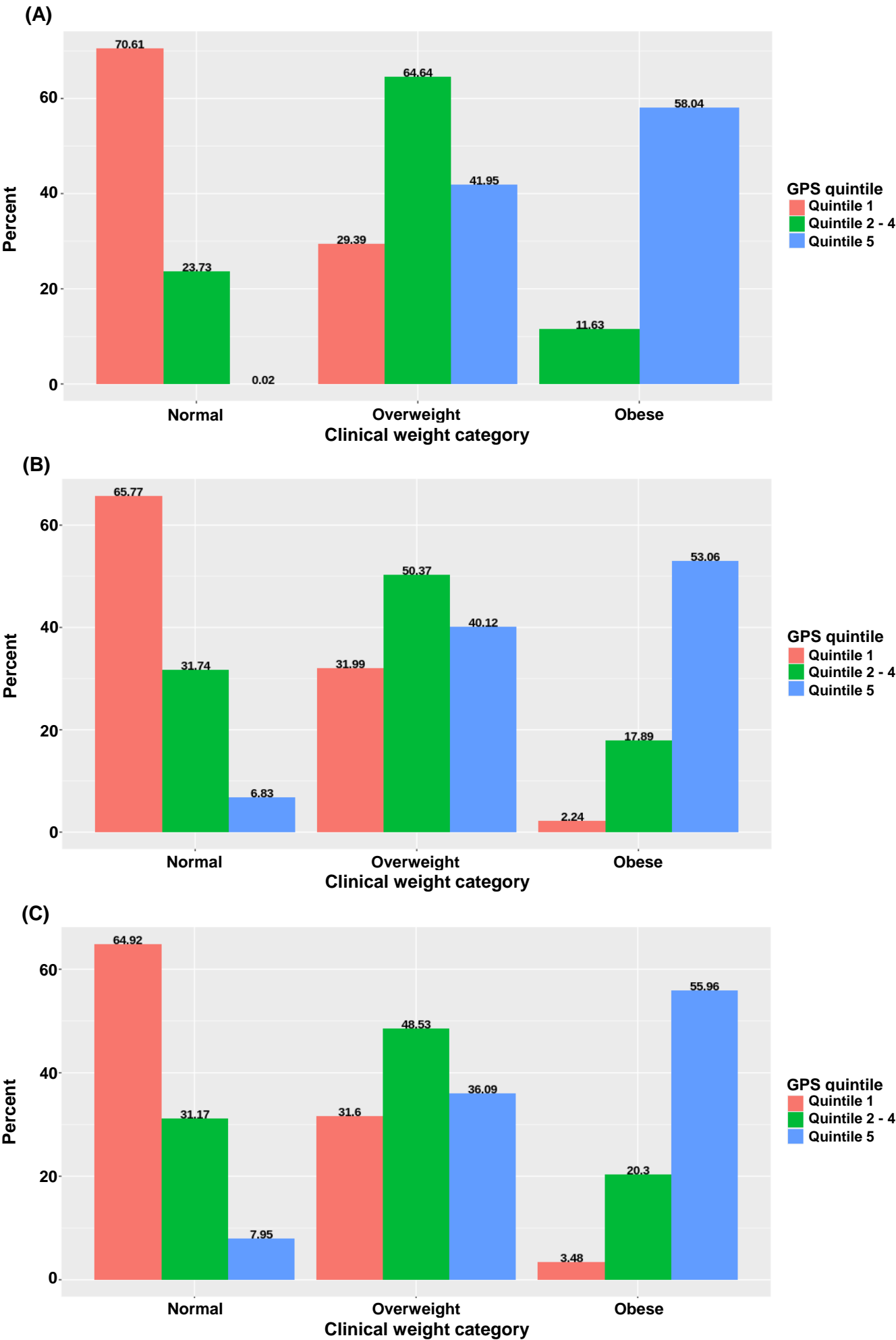

Supplement: Supplementary file 1 [file DataSheet1.PDF]
